# Characterization of Nuclear Localization and SUMOylation of the ATBF1 Transcription Factor in Epithelial Cells

**DOI:** 10.1371/journal.pone.0092746

**Published:** 2014-03-20

**Authors:** Xiaodong Sun, Jie Li, Frederick N. Dong, Jin-Tang Dong

**Affiliations:** Winship Cancer Institute, Department of Hematology and Medical Oncology, Emory University School of Medicine, Atlanta, Georgia, United States of America; The University of Hong Kong, Hong Kong

## Abstract

ATBF1/ZFHX3 is a large transcription factor that functions in development, tumorigenesis and other biological processes. ATBF1 is normally localized in the nucleus, but is often mislocalized in the cytoplasm in cancer cells. The mechanism underlying the mislocalization of ATBF1 is unknown. In this study, we analyzed the nuclear localization of ATBF1, and found that ectopically expressed ATBF1 formed nuclear body (NB)-like dots in the nucleus, some of which indeed physically associated with promyelocytic leukemia (PML) NBs. We also defined a 3-amino acid motif, KRK^2615-2617^, as the nuclear localization signal (NLS) for ATBF1. Interestingly, diffusely distributed nuclear SUMO1 proteins were sequestered into ATBF1 dots, which could be related to ATBF1's physical association with PML NBs, known SUMOylation hotspots. Furthermore, ATBF1 itself was SUMOylated. ATBF1 SUMOylation occurred at more than 3 lysine residues including K^2349^, K^2806^ and K^3258^ and was nuclear specific. Finally, the PIAS3 SUMO1 E3 ligase, which interacts with ATBF1 directly, diminished rather than enhanced ATBF1 SUMOylation, preventing the co-localization of ATBF1 with SUMO1 in the nucleus. These findings suggest that nuclear localization and SUMOylation are important for the transcription factor function of ATBF1, and that ATBF1 could cooperate with PML NBs to regulate protein SUMOylation in different biological processes.

## Introduction

The AT-motif binding factor 1/zinc finger homeobox 3 (ATBF1/ZFHX3) is a 404-kD transcription factor containing four homeodomains and multiple zinc-finger motifs [1]. It functions in multiple biological processes including embryonic development [2], mammary gland development [3], neuronal differentiation [4]-[6], and neuronal death in response to DNA damage or oxidative stress [7], [8]. For example, loss of a single allele of the *Atbf1* gene in mice results in severe preweaning mortality and partial embryonic lethality [2]. ATBF1 abnormalities play a role in multiple human diseases including tumorigenesis [9], [10], Kawasaki disease (KD), and atrial fibrosis [11]–[13]. For example, *ATBF1* is the second most frequently mutated gene in human prostate cancer [9], [14], its expression is frequently reduced in multiple types of cancers [15]–[19], and tissue-specific deletion of *Atbf1* in mouse prostates causes neoplastic alterations (Sun et al., manuscript submitted).

ATBF1 was originally identified as a transcriptional repressor of alpha-fetoprotein (AFP) [20], and a number of studies have demonstrated that ATBF1 interacts with other transcription factors to regulate the transcription of many genes [21], including those involved in enterocyte and myogenic differentiation and early development of the pituitary gland [22]–[24], and those that encode for membrane and secretory proteins (Sun et al., manuscript submitted). As expected for a transcription factor, ATBF1 is localized in the nucleus [3], [5], [10], [19]. In human breast, gastric, skin, head and neck and possibly other cancers however, ATBF1 is often mislocalized to the cytoplasm, and the mislocalization is associated with histopathologic progression and worse patient survival [10], [15], [19]. A higher nuclear ATBF1 level was also associated with lower expression of oncogenic MUC5AC and a better prognosis in gastric cancer [25], [26].

While a previous study has demonstrated that ATBF1 translocates to the nucleus with RUNX3 in response to TGFβ stimulation in gastric cancer cells [19], the mechanisms controlling the cellular localization of ATBF1 remain to be illustrated, and whether posttranslational modifications of ATBF1 depend on or determine its nuclear localization is unknown. While ATBF1 can be phosphorylated at multiple serine residues during DNA damage response or brain development [27], [28] and modified by polyubiquitination at lysines [29], both of which affect ATBF1 stability, it is unknown whether ATBF1 undergoes other posttranslational modifications.

By characterizing the nuclear localization of ATBF1 in this study, we found that ectopically expressed ATBF1 formed nuclear body (NB)-like dots in the nucleus of epithelial cells, and its nuclear localization was mediated by a 3-amino acid motif. Interestingly, ATBF1 dots were associated with one of the most common NBs seen in mammalian cells, promyelocytic leukemia (PML) NBs. Possibly related to the SUMOylation function of PML NBs, ATBF1 sequestered diffusely distributed SUMO1 into ATBF1 dots, and the sequestration was interrupted by PIAS3, an ATBF1-interacting SUMOylation E3 ligase. Furthermore, ATBF1 itself was also SUMOylated in the nucleus at more than 3 lysine residues, and ATBF1 SUMOylation was unexpectedly negatively affected by PIAS3.

## Materials and Methods

### Cell lines

Prostate cancer cell line 22Rv1 was obtained from the ATCC (Manassas, VA) and maintained in RPMI-1640 medium following the ATCC's instructions.

### Plasmids

The original *ATBF1* cDNA was obtained from Dr. Yutaka Miura (Nagoya City University, Nagoya, Japan). An inframe deletion of 24 nucleotides in the original cDNA, which is associated with prostate cancer risk [30], was patched with a DNA fragment from the I.M.A.G.E. clone 3538674. The 5′ and the 3′ termini were further engineered by introducing the SalI recognition sequence. Then the full length *ATBF1* cDNA was subcloned into the *pKXU-HA* and *pEGFP-C3* vectors to generate *HA*-tagged and *EGFP*-fused *ATBF1* constructs, respectively. Clones containing the *ATBF1* cDNA in the antisense direction were also obtained and named antisense-*ATBF1*. Other *ATBF1* gene fragments were obtained either by restriction deoxyribonuclease digestion of the full-length *ATBF1* cDNA or by PCR amplification, and the resultant cDNA fragments were inserted into the *pEGFP-C3* or *pKXU-HA* vectors. To generate a full length *ATBF1* mutant, *ATBF1* fragments were subcloned into a modified *pBlueScript SK+* vector, and point mutations were generated by PCR-driven overlap extension [31]. After confirmation by DNA sequencing, mutant fragments were used to replace the corresponding fragments in the full length wildtype *ATBF1* cDNA plasmid.

*Myc-Ubc9* and *GFP-SUMO1* plasmids were kindly provided by Dr. Jihe Zhao (Albany Medical College) and Dr. Elliott Kieff (Harvard University), respectively. The human *PIAS3* cDNA was purchased from Origene (Rockville, MD) and was subcloned into the *FLAG-pcDNA3* vector. All other constructs were generated by the PCR approach and subcloned into the *pcDNA3*, *FLAG-pcDNA3*, or *pEGFP-C3* vectors.

### Antibodies

The anti-ATBF1 polyclonal antibody was kindly provided by Dr. Yutaka Miura. Other primary antibodies used in this study included rabbit anti-HA, rabbit anti-FLAG, mouse anti-FLAG, and mouse anti-actin antibodies, which were from Sigma-Aldrich (St. Louis, MO); rabbit anti-SUMO1 and mouse anti-PML antibodies from Santa Cruz (Dallas, TX); and rabbit anti-Myc polyclonal antibody from Delta Biolabs (Gilroy, CA).

### Immunofluorescence

Cells were cultured in 4-well chamber slides for 24 hours prior to plasmid transfection using the Lipofectamine Plus reagent (Invitrogen, Carlsbad, CA). Forty-eight hours after transfection, cells were fixed with 4% paraformaldehyde in phosphate-buffered saline (PBS) for 10 min at room temperature, followed by permeabilization and blockage in PBS containing 10% (v/v) normal goat serum and 0.3% (v/v) Triton X-100 for 30 min. Cells were then incubated with the primary antibodies at 4°C overnight and washed three times for 10 min each in PBS. After washing, cells were incubated with an Alexa Fluor fluorochrome-conjugated secondary antibody (Invitrogen, Carlsbad, CA) for 60 min at room temperature. After three washes in PBS, nuclei were counterstained with 4′,6-diamidino-2-phenylindole (DAPI) for 5 min. The chamber on each slide was then removed, and slides were mounted and visualized under a confocal laser microscope (Zeiss, Oberkochen, Germany).

### Protein immunoprecipitation and immunoblotting assays

For immunoprecipitation, 22Rv1 cells were grown to 50–70% confluence in 10-cm dishes and each dish was transfected with 4 μg of total plasmids using the Lipofectamine Plus reagent (Invitrogen). At 48 hours post-transfection, cells were washed twice with cold PBS, and then lysed at 4°C for 30 min by gentle shaking in 0.5 ml of NP-40 cell lysis buffer (150 mM NaCl, 10 mM Tris - pH 7.5, 0.2% Nonidet P-40, 5 mM NaPyrophosphate, 5 mM NaF and 2 mM NaOrthovanadate) supplemented with 1% protease inhibitor cocktail and 20 mM N-ethylmaleimide (NEM) (Sigma-Aldrich, St Louis, MO). After centrifugation at 10,000 g for 10 min, the supernatant was collected and measured for protein concentration by the Bradford assay (Bio-Rad Laboratories, Hercules, CA). Fifty μg of protein lysate were kept as the input control, while 500 μg were incubated with 30 μl of anti-HA beads (Sigma-Aldrich) for 2 hours. The beads were washed with cold NP-40 lysis buffer three times, and immunoprecipitates were eluted with lysis buffer by heating at 95°C for 5 min.

For immunoblotting (IB), cell lysates or immunoprecipitates were separated by 4% (for full length ATBF1) or 10–12% (for all other proteins) SDS-polyacrylamide gel electrophoresis (PAGE), and electrophoretically transferred to nitrocellulose membranes, which were then blocked with 5% fat-free milk and incubated with the primary antibodies. The membranes were then incubated with horseradish peroxidase-conjugated anti-IgG secondary antibodies (Sigma-Aldrich) and visualized with an enhanced chemiluminescence system (Pierce Protein Research Products, Thermo Scientific, Rockford, IL).

### *In vitro* translation and SUMOylation assay

For *in vitro* protein translation, PCR products of wildtype and mutant ATBF1 cDNA fragments were amplified by PCR according to the requirements of the TNT T7 Quick *in vitro* synthesis kit (Promega, Madison, WI). PCR products were purified, mixed with ^35^S-methionine and the Master Mix from the kit, and incubated for 1 hour at 30°C for transcription and translation to occur, generating ATBF1 protein fragments for the *in vitro* SUMOylation assay. The *in vitro* SUMOylation kit was purchased from LAE Biotechnology (Rockville, MD). Following the manufacturer's instructions, the assays were carried out in a final volume of 20 μl in a reaction buffer containing 20 mM HEPES pH 7.5, 5 mM MgCl_2_ and 2 mM ATP. *In vitro* translated proteins were added with 1 μg of E2, 1 μg of SUMO1 (active form), 150 ng of E1, and 4 mM ATP sequentially. The control reactions contained the same components except for SUMO1. After 90 min of incubation at 37°C, reactions were denatured in the sample loading buffer and separated by 15% SDS-PAGE. Gels were dried and subjected to autoradiography overnight.

## Results

### ATBF1 forms nuclear body (NB)-like dots in the nucleus

**Figure 1:**
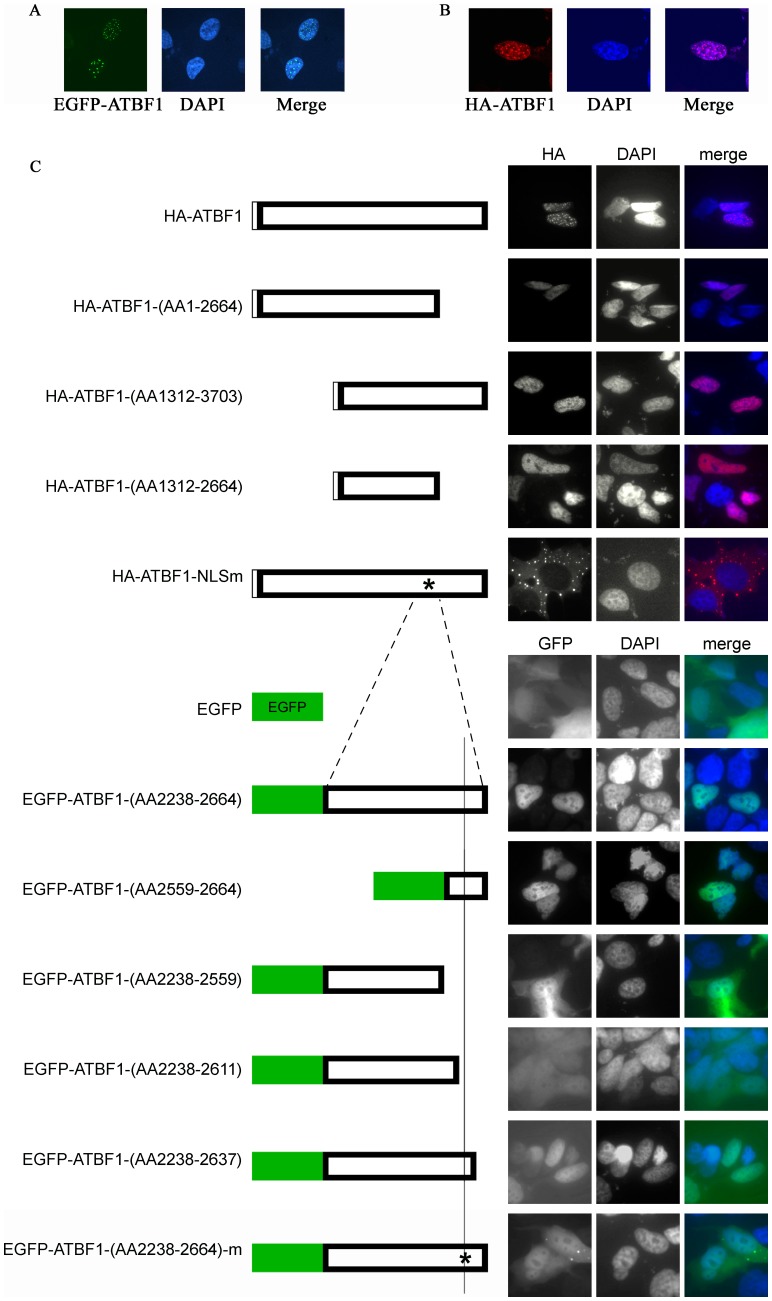
Detection of nuclear body (NB)-like ATBF1 dots in the nucleus and definition of the ATBF1 nuclear localization signal (NLS). A, B. ATBF1 concentrates to form NB-like dots in the nucleus, as detected by immunofluorescent microscopy in 22Rv1 cells expressing EGFP-fused ATBF1 (A) or HA-tagged ATBF1 (B). C. Identification of the NLS for ATBF1 by deletion mapping, mutation and immunofluorescent microscopy. Each box at left represents a fragment of ATBF1 or the full length ATBF1 (wildtype or mutant as indicated by *) attached with the EGFP or the HA tag. Each fragment box is aligned to full length ATBF1 to indicate its relative location in ATBF1. Images at right indicate the subcellular localization of a fragment or full length ATBF1, with the nucleus shown by DAPI staining. Residue numbers are based on the ATBF1-A protein sequence (NCBI access number NP_008816).

To characterize the nuclear localization of ATBF1 in epithelial cells, we expressed EGFP-fused ATBF1 in 22Rv1 prostate cancer cells, which express only scarce endogenous ATBF1, and detected EGFP-ATBF1 protein by microscopy. Whereas EGFP alone was diffusely distributed in both the nucleus and the cytoplasm, EGFP-ATBF1 was only detected in the nucleus, which was visualized by DAPI staining (Fig. 1A). Surprisingly, EGFP-ATBF1 proteins massed to form nuclear body-like dots (ATBF1 dots) in the nucleus (Fig. 1A). To rule out an effect of EGFP fusion on ATBF1 localization, we also expressed HA-tagged ATBF1 in 22Rv1 cells, and found the same result: nuclear ATBF1 formed dots (Fig. 1B).

### Nuclear localization of ATBF1 depends on its nuclear localization signal (NLS)

We then performed a series of deletion mapping, ectopic expression and immunofluorescence imaging with both EGFP-fused ATBF1 and HA-tagged ATBF1 to identify the nuclear localization signal (NLS) of ATBF1. A number of *ATBF1* fragments were generated and transfected into 22Rv1 cells. As shown in Figure 1C, deletion of either the N-terminal amino acids (AA) 1-1311 or the C-terminal AA2665-3703 of ATBF1 did not affect its nuclear localization, limiting the NLS to the middle region AA1312-2664 of ATBF1. Deletion of AA 2238-2664 from the middle region abolished its nuclear localization (data not shown), and the EGFP-fused fragment AA2238-2664 localized into the nucleus, indicating that the NLS is located within AA2238-2664 of ATBF1 (Fig. 1C). Further deletions within the EGFP-ATBF1-(AA2238-2664) fragment and localization analyses demonstrated that the NLS is located in a small 27-residue fragment – AA2611-2637, because deletion of this 27-AA fragment attenuated the nuclear localization of ATBF1 (Fig. 1C).

Within the 27-AA sequence, while no classical NLS sequence was found, a 3-AA sequence (Lys-Arg-Lys) (KRK^2615–2617^) harbored positive charges that could facilitate the transfer of a protein through nuclear pores. We therefore mutated these three amino acids (KRK^2615–2617^) into alanines (AAA^2615–2617^), and examined whether the mutation affects ATBF1's nuclear localization. This mutation indeed prevented both EGFP-fused ATBF1 and HA-tagged ATBF1 from entering the nucleus (Fig. 1C), confirming that the KRK^2615–2617^ sequence is the NLS for ATBF1.

### Association of nuclear ATBF1 with PML nuclear bodies (NBs)

**Figure 2:**
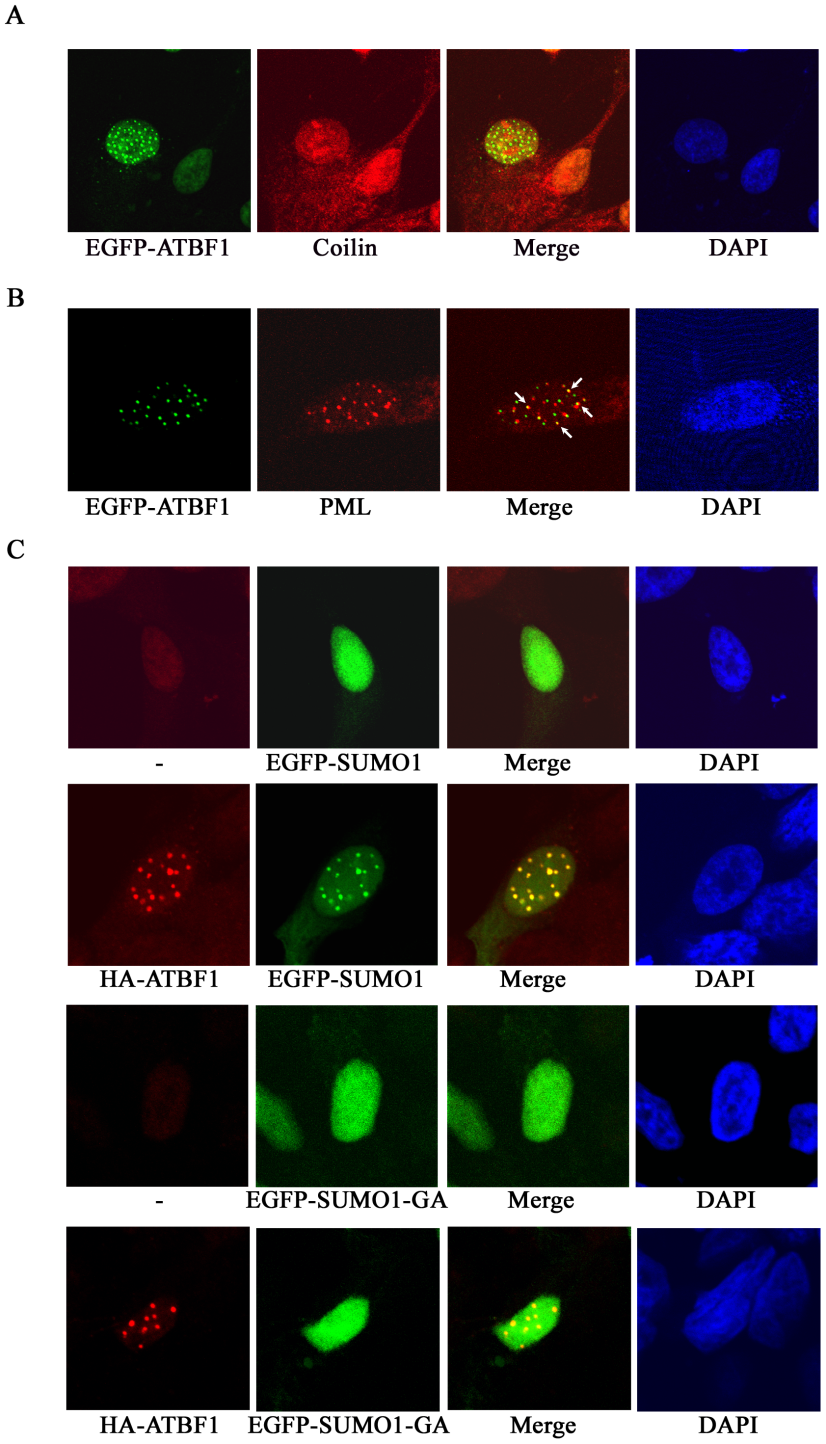
Association of ATBF1 with PML nuclear bodies (PML NBs) and SUMO1 in the nucleus, as detected by immunofluorescent microscopy in 22Rv1 cells. A, B. ATBF1 dots are not associated with Cajal bodies (A) but partially overlap with a subset of PML NBs (arrows) (B). ATBF1 dots were visualized by EGFP (green), and Cajal bodies and PML NBs by an anti-Coilin or anti-PML antibody, respectively (red). C. Co-localization of ATBF1 and SUMO1. ATBF1 dots were detected by an anti-HA antibody (red), and wild type and mutant SUMO1 proteins by EGFP (green). While wildtype SUMO1 is co-localized with ATBF1 dots, the mutant form (EGFP-SUMO1-GA) is not. Nuclei are shown by DAPI counterstaining.

A number of nuclear bodies such as Cajal bodies and PML nuclear bodies (PML NBs) have been described in mammalian cells [32], [33]. We therefore evaluated whether ATBF1 dots have a relationship with these known nuclear structures by expressing EGFP-fused ATBF1 in 22Rv1 cells and staining the cells with anti-coilin and anti-PML antibodies. Coilin is a key member of Cajal bodies whereas PML is the essential organizer of PML NBs [32], [33], so comparing the localization of ATBF1 dots with coilin dots or PML dots would indicate their relationships. Whereas ATBF1 dots did not show any association with Cajal bodies (Fig. 2A), some ATBF1 dots either partially overlapped or were closely associated with PML NBs (Fig. 2B). These results indicate the existence of a spatial relationship between some ATBF1 dots and some PML-NBs.

### ATBF1 associates with and alters the distribution of SUMO1 in the nucleus

PML nuclear bodies (NBs) are nuclear matrix domains that serve as a scaffold for PML-NB-interacting proteins to undergo posttranslational modifications [33], [34]. A large number of proteins that interact with PML NBs have been identified. The most common feature shared among these partner proteins is their SUMOylation, although other posttranslational modifications such as ubiquitination, phosphorylation and acetylation also occur [33], [35]–[37]. Furthermore, an E3 ligase of SUMOylation, the protein inhibitor of activated STAT3 (PIAS3), was previously shown to directly interact with ATBF1 [38]. We therefore tested whether ATBF1 is associated with SUMO1 or has any effect on SUMO1 distribution. We transfected expression plasmids for EGFP-fused ATBF1 and/or SUMO1 into 22Rv1 cells, and visualized the proteins by immunofluorescent microscopy. Without the co-expression of ATBF1, SUMO1 protein was diffusely distributed in the nucleus and few speckles of SUMO1 were visible (Fig. 2C). When EGFP-ATBF1 was co-expressed however, SUMO1 proteins were highly concentrated into a number of speckles in the nucleus, and surprisingly, these SUMO1 speckles overlapped with ATBF1 dots (Fig. 2C). When the SUMOylation-deficient form of SUMO1, SUMO1-GA, was co-expressed, which is unable to conjugate to target proteins due to the mutation, SUMO1 failed to form speckles and co-localize with ATBF1 dots, although the mutant SUMO1-GA alone was still diffusely distributed in the nucleus (Fig. 2C). These results suggest that ATBF1 interacts with SUMO1 to alter its nuclear distribution.

### ATBF1 itself is SUMOylated at multiple lysine residues

**Figure 3:**
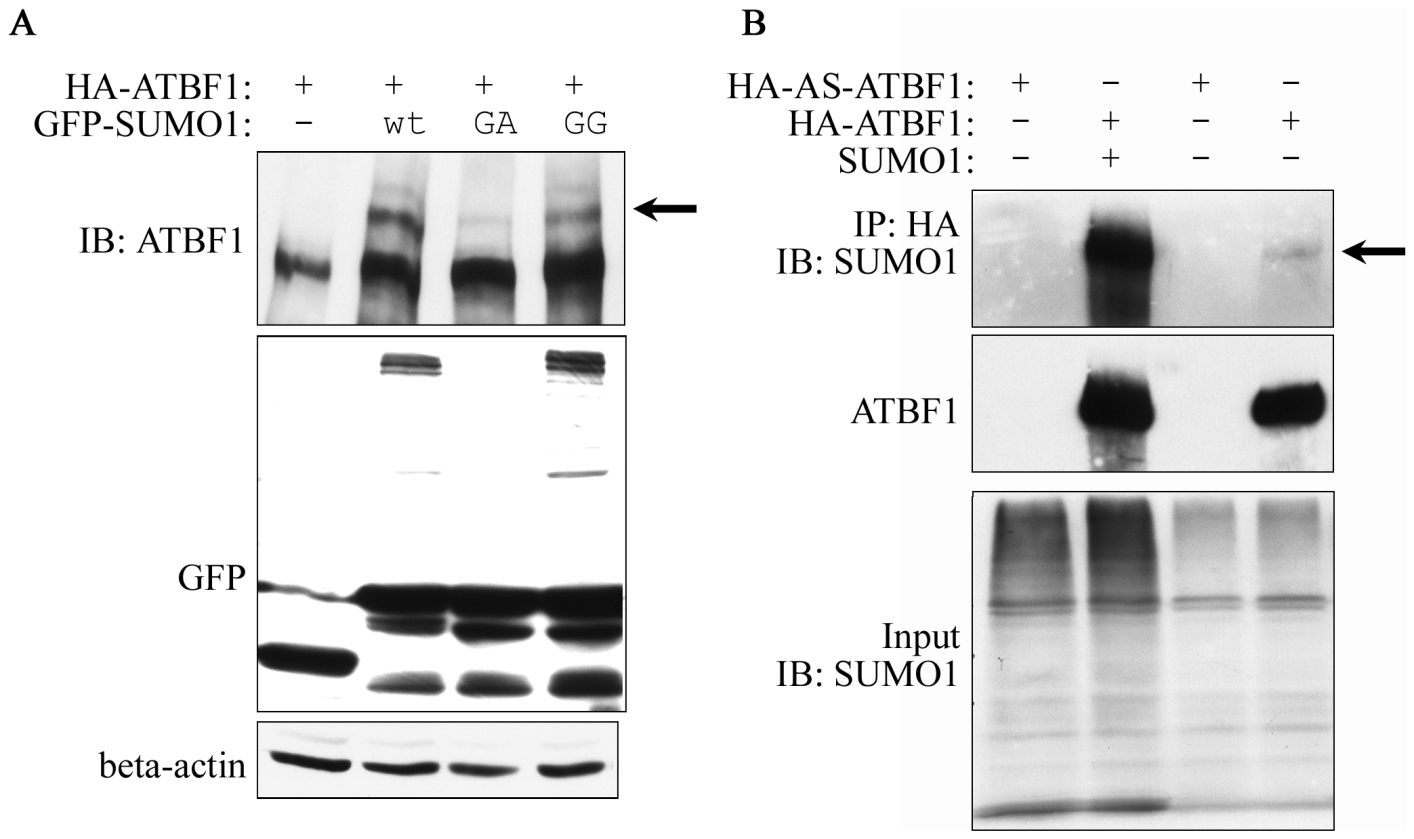
Detection of ATBF1 SUMOylation by IB (A) and immunoprecipitation combined with IB (B). A. Both native and SUMOylated ATBF1 were detected by the anti-ATBF1 antibody. SUMO1 was detected by anti-GFP antibody. B. ATBF1 was pulled down by anti-HA beads, and eluted proteins were blotted with anti-SUMO1 or anti-ATBF1 antibody. The faint band in the upper panel (far right lane) indicates ATBF1 SUMOylation by endogenous SUMO1. The arrows in A and B indicate the shifted ATBF1 band, representing SUMOylated ATBF1. The native ATBF1 protein is about 400 kD. Plasmids used for transfection are listed at the top, including HA tagged ATBF1 (HA-ATBF1) and antisense ATBF1 (HA-AS-ATBF1).

Based on the findings of ATBF1-SUMO1 association (Fig. 2C), we then tested whether ATBF1 itself is covalently modified by SUMO1. EGFP-fused SUMO1 expression plasmid, along with the expression vector pEGFP-C3 as the control, was co-transfected into 22Rv1 cells with HA-tagged ATBF1 expression plasmid, and cell lysates were then subjected to IB to determine whether SUMO1 expression can shift the ATBF1 band in a gel, which is an indicator of protein SUMOylation. Compared to the vector control, EGFP-SUMO1 expression caused the appearance of two larger ATBF1 bands in addition to the major one (Fig. 3A). The two larger ATBF1 bands were only detected with the active form of SUMO1 (EGFP-SUMO1-GG) but not with the SUMOylation-deficient form of SUMO1, EGFP-SUMO1-GA (Fig. 3A).

We then performed co-IP combined with IB to determine whether ATBF1 is indeed SUMOylated. HA-tagged sense- or anti-sense-ATBF1 was co-transfected with the plasmid pcSUMO1, which expresses full length SUMO1 without any tag. ATBF1 was pulled down by co-IP with anti-HA antibody beads, and then subjected to IB with either anti-HA or anti-SUMO antibody. Both SUMO1 and ATBF1 signals were detected at the same position in the gel (Lane 2 in Fig. 3B), indicating the SUMOylation of ATBF1. Even in the sample without the co-transfection of SUMO1 plasmid, a faint band was also detected by the anti-SUMO1 antibody at the ATBF1 location in the gel, suggesting that ATBF1 SUMOylation occurs with endogenous SUMO1 (Fig. 3B).

**Figure 4:**
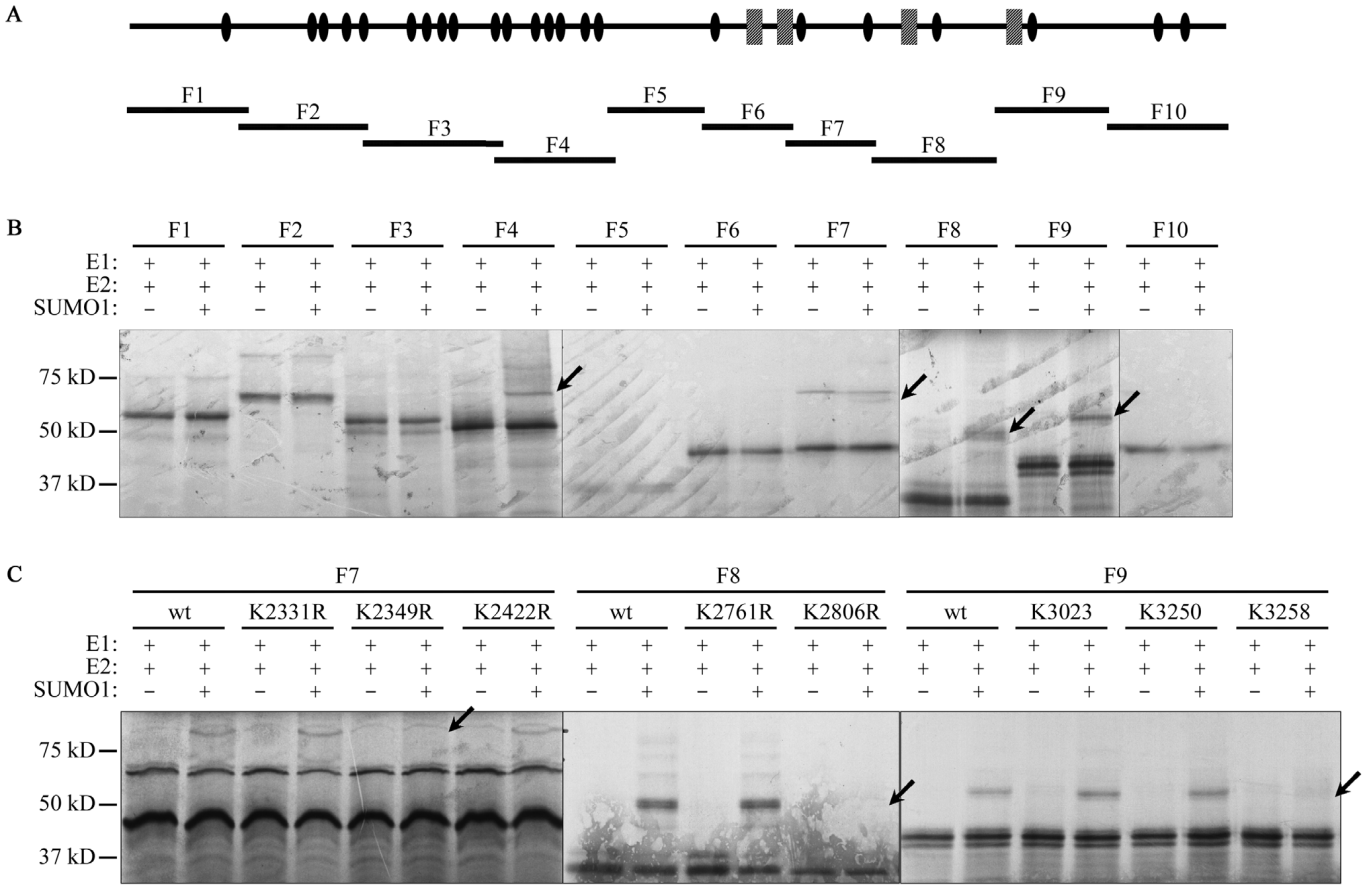
Identification of SUMOylation sites of ATBF1. A. Location of 10 ATBF1 fragments (F1-F10) relative to the full-length ATBF1 protein (top) for the *in vitro* SUMOylation assay. In the schematic for full-length ATBF1, potential functional domains, including zinc fingers (ovals) and homeodomains (rectangles), are shown based on previous predictions by Miura et al. [1]. B. Detection of SUMOylated ATBF1 fragments *in vitro*. For each fragment, SUMO1 was present (+) or absent (-) in the reactions. Arrows point to SUMOylated ATBF1 fragments. C. Identification of lysine residues that are SUMOylated in the 3 ATBF1 fragments. *In vitro* SUMOylation assay was performed for ATBF1 fragments with different lysine mutants. Arrows indicate the disappearance of SUMOylated ATBF1 peptides in the K2349R, K2806R and K3258R mutations of the 3 fragments.

We then attempted to identify the amino acids in ATBF1 that are SUMOylated. SUMOylation usually occurs at the lysine residue(s) of target proteins, and ATBF1 protein contains 225 lysines. We divided the full length ATBF1 into 10 overlapping fragments, and performed *in vitro* translation and SUMOylation assay for each fragment (Fig. 4A). As shown in Figure 4B, fragments F4, F7, F8 and F9 of ATBF1 showed modification when incubated with SUMO enzymes and active SUMO1, indicating potential SUMOylation sites in these fragments. We then mutated several potential lysine residues in these four fragments into arginines, and performed *in vitro* translation and SUMOylation assays again to identify the lysines that are SUMOylated. Mutations of lysine residues at 2349, 2806 and 3258 (K2349R, K2806R and K3258R) in F7, F8, and F9, respectively, abolished the *in vitro* SUMOylation of the respective fragment, indicating that these three lysines are the target residues for SUMO1 modification (Fig. 4C). Among these three lysine residues, K2806 and K3258 are in the ψKXE consensus SUMOylation sequence (IK^2806^VE and PK^3258^KE) [39] while K2349 is not. Using the same method, we were unable to identify the lysine residue for SUMOylation in the F4 fragment (data not shown).

### ATBF1 SUMOylation is nuclear specific

**Figure 5:**
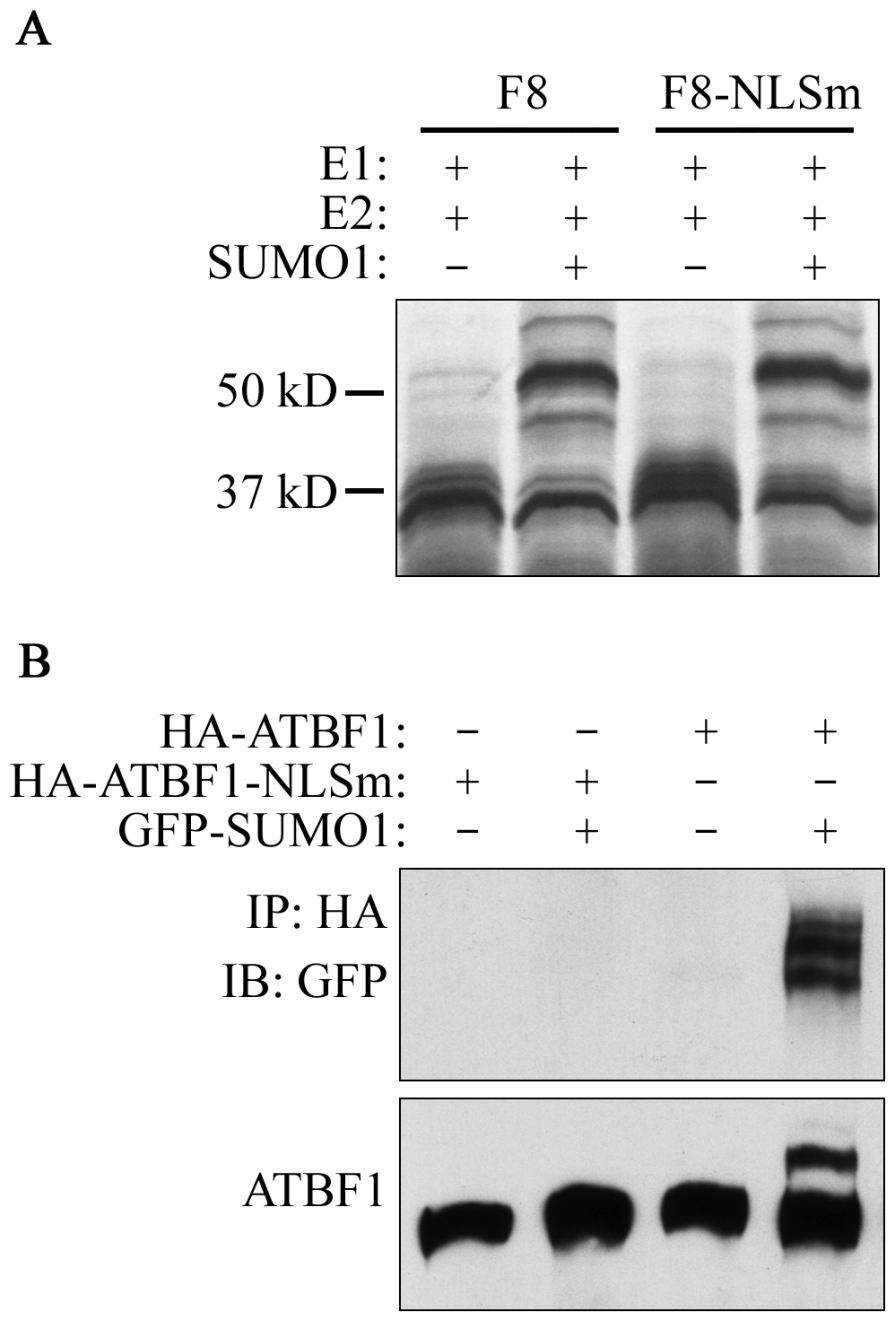
Nuclear localization is essential for ATBF1 to undergo SUMOylation. A. Mutation of the NLS at residues KRK^2615–2617^ had no effect on the SUMOylation of ATBF1 fragment 8 in the *in vitro* assay, as detected by IB. B. Failure in nuclear localization prevents the SUMOylation of ATBF1. HA-tagged wildtype ATBF1 (HA-ATBF1) or a mutant that was deficient in the NLS (HA-ATBF1-NLSm) were co-transfected with GFP-tagged SUMO1 into 22Rv1 cells, and cell lysates were pulled down with anti-HA beads, and blotted with anti-GFP (upper) or anti-ATBF1 antibody (lower) to detect SUMOylated ATBF1. The native ATBF1 protein is about 400 kD.

SUMOylation modification occurs in either the nucleus or the cytoplasm. For some nuclear proteins, SUMOylation affects their nuclear transportation [39]. We therefore determined whether ATBF1 SUMOylation occurs in the nucleus or in the cytoplasm. The NLS Lys-Arg-Lys (KRK^2615–2617^) is located in the F8 fragment of ATBF1, so we first mutated KRK^2615–2617^ into AAA^2615–2617^ in F8 and analyzed the SUMOylation of the mutant F8 fragment *in vitro*. SUMOylated F8 was still detected in the *in vitro* SUMOylation assay (Fig. 5A), indicating that mutation of the NLS of ATBF1 does not affect its SUMOylation. We then transfected HA-tagged wildtype full-length ATBF1 (HA-ATBF1) or the NLS-deficient mutant of ATBF1 (HA-ATBF1-NLSm) with EGFP-SUMO1 into 22Rv1 cells, and determined whether nuclear localization has an effect on ATBF1 SUMOylation. ATBF1 was first pulled down with anti-HA antibody beads and then blotted for either SUMOylation with anti-GFP antibody or for ATBF1 with anti-HA antibody. SUMOylated ATBF1 was only detected with the wildtype ATBF1 but not with the NLS-deficient ATBF1 (Fig. 5B), indicating that nuclear localization is essential for ATBF1 to undergo SUMOylation.

### PIAS3 attenuates ATBF1 SUMOylation

**Figure 6:**
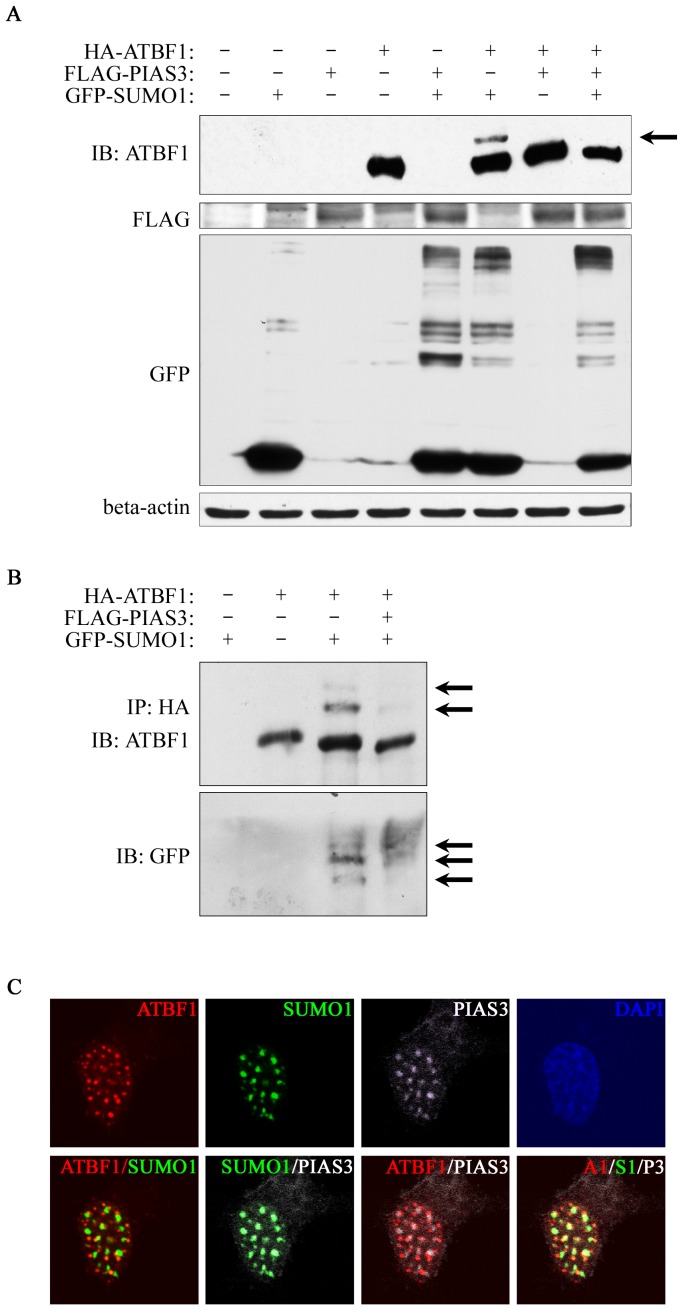
PIAS3 interrupts ATBF1 SUMOylation. A. PIAS3 reduces ATBF1 SUMOylation as detected by IB. Plasmids used for transfection are listed at the top. ATBF1, PIAS3 and SUMO1 were detected by anti-ATBF1, anti-FLAG and anti-GFP antibodies respectively. The arrow indicates SUMOylated ATBF1. The native ATBF1 protein is about 400 kD. B. Confirmation of PIAS3-mediated reduction in ATBF1 SUMOylation by co-immunoprecipitation and IB. ATBF1 was pulled down by anti-HA beads, and eluted proteins were blotted with anti-ATBF1 and anti-SUMO1 antibodies sequentially. Arrows indicate bands of SUMOylated ATBF1. C. Expression of PIAS3 prevents the co-localization of SUMO1 with ATBF1. Immunofluorescence staining was used to detect PIAS3 (anti-FLAG antibody, white), SUMO1 (EGFP, green), and ATBF1 (anti-HA antibody, red). Merged images between any two of the molecules or among all three (A1/S1/P3) are shown on the lower panels. Nuclei were counterstained with DAPI.

Although SUMOylation E3 ligases are not required for efficient SUMOylation, they usually promote SUMOylation of target proteins [40]. One such E3 ligase, PIAS3, interacts with ATBF1 [38], so we tested whether the ATBF1-PIAS3 interaction affects the SUMOylation of ATBF1. Using the same approaches of co-expressing HA-ATBF1 and EGFP-SUMO1 and IB as in Figure 3A, where SUMOylated ATBF1 bands could be clearly detected, we found that co-expression of PIAS3 diminished rather than enhanced SUMOylated ATBF1 bands (Fig. 6A). We also pulled down ATBF1 protein by co-IP with anti-HA antibody and detected ATBF1 with anti-ATBF1 antibody, and confirmed that PIAS3 expression diminished the extra SUMOylated bands of ATBF1 (Fig. 6B).

We also co-expressed ATBF1, SUMO1 and PIAS3 in 22Rv1 cells and evaluated the effect of PIAS3 expression on the co-localization of ATBF1 and SUMO1 by immunofluorescent microscopy. Consistent with the biochemical findings (Fig. 6A, 6B), expression of PIAS3 prevented the colocalization of ATBF1 with SUMO1 (Fig. 6C). Furthermore, when PIAS3 was expressed, both PIAS3 and SUMO1 formed punctate structures, and those of SUMO1 predominantly co-localized with those of PIAS3 (Fig. 6C). Whereas ATBF1 dots were not overlapping with the SUMO1-PIAS3 dots, some ATBF1 dots were adjacent to the SUMO1-PIAS3 dots (Fig. 6C). These biochemical and cellular results indicate that, instead of promoting ATBF1 SUMOylation, PIAS3 interrupts both ATBF1 SUMOylation and the association of ATBF1 dots with SUMO1 dots.

## Discussion

ATBF1 concentrates to form nuclear body-like dots in the nucleus, and its nuclear localization is mediated by a 3-AA motif KRK^2615–2617^. As a transcription factor, ATBF1 is expected to be located in the nucleus to function, and its nuclear localization has been confirmed in both cultured cells and human tissues [3], [10], [41]. It has also been demonstrated that in the nucleus, ATBF1 interacts with other nuclear factors to regulate gene expression and cell proliferation [21], [42]. Nuclear localization is expected to be important for ATBF1 function because mislocalization of ATBF1 into the cytoplasm occurs in cancer cells and is associated with worse survival in cancer patients [10], [15], [19], [26]. Whereas in gastric cancer cells ATBF1 is located in the cytoplasm and can be tanslocated into the nucleus with RUNX3 upon TGFβ activation [19], we found that ectopically expressed ATBF1 is localized in the nucleus in 22Rv1 prostate cancer cells (Fig. 1) as in normal cells. Interestingly, ATBF1 formed nuclear body-like protein dots in the nucleus (Fig. 1). In addition, we identified a 3-AA motif, Lys-Arg-Lys at residues 2615–2617 (KRK^2615–2617^) as the NLS that mediates the nuclear localization of ATBF1, because mutations of these residues prevented ATBF1 from entering the nucleus (Fig. 1). The sequence of ATBF1's NLS does not match any previously predicted NLS sequences [5].

ATBF1 dots associate with PML NBs, and this association may be involved in protein SUMOylation. The two most common NBs in mammalian cells are PML NBs and Cajal bodies [32], [33]. Whereas the NB-like ATBF1 dots had no detectable association with Cajal bodies, they were associated with PML NBs, since some ATBF1 dots overlapped with some PML NBs (Fig. 2). Although definitive evidence is lacking at this time, the association of ATBF1 dots with PML NBs could have functional implications. For example, PML NBs function as nuclear SUMOylation hotspots, and a large number of PML-NB-interacting proteins are also directly SUMOylated, which is the most common feature among these proteins [33], [35]–[37], [43]. In addition, PML NBs themselves are also regulated by SUMOylation at two levels. Firstly, PML is directly SUMOylated at multiple lysine residues, and SUMOylation is essential for the formation of PML NBs [34], [44], [45]. Secondly, PML also has a SUMO binding motif that is independent of its SUMOylation sites but is also necessary for PML NB formation [35]. On the other hand, ATBF1 expression induced the aggregation of SUMO1 into ATBF1 dots (Fig. 2), while ATBF1 did not appear to directly interact with SUMO1 (data not shown) and the PIAS3 SUMO E3 ligase altered the association between ATBF1 dots and SUMO1 dots (Fig. 6), suggesting that ATBF1 could also play a role in the SUMOylation of other proteins. The fact that ATBF1 itself was SUMOylated at multiple lysine residues (Fig. 2–4) could also be related to the role of ATBF1 in the regulation of protein SUMOylation, as seen for PML NBs. The association of ATBF1 dots with PML NBs thus suggests that ATBF1 could cooperate with PML NBs to regulate protein SUMOylation. While this remains to be tested, there are published studies that appear to support this possibility. For example, ATBF1 could cooperate with PML NBs to regulate activities of the Myb oncoprotein, because Myb is subjected to SUMOylation for activity regulation, it localizes to PML NBs via direct interaction with PML, and it also directly interacts with ATBF1 to regulate its activity [42], [46]. As a large protein that associates with PML NBs, ATBF1 could be part of the PML NB scaffold for protein SUMOylation to occur. It should thus be meaningful to directly test whether ATBF1 cooperates with PML NBs to regulate protein SUMOylation and activities.

ATBF1 itself is SUMOylated at multiple lysine residues. As a large transcription factor (∼404 kD), it is anticipated that ATBF1 undergoes different types of post-translational modifications. However, only phosphorylation of several serine residues and ubiquitination have been reported thus far for ATBF1 [28], [29]. Our findings in this study indicate that ATBF1 is covalently modified by SUMO1 at multiple lysine residues, including K^2349^, K^2806^ and K^3258^ (Fig. 2–4). We applied three approaches to confirm the SUMOylation of ATBF1, including co-localization of ATBF1 and SUMO1 in the nucleus (Fig. 2), detection of SUMO1-conjugated ATBF1 by IB (Fig. 3), and identification of multiple SUMOylation sites in the ATBF1 protein (Fig. 4). While two of the three SUMOylation sites, K^2806^ and K^3258^, are in the typical SUMOylation consensus motif (ψKXE), one (K^2349^) is not. A growing number of proteins have been identified that are SUMOylated at non-consensus sequences, including SMAD4, CREB, PCNA and Daxx [47]-[50], which causes difficulties in identifying SUMOylation sites in large proteins such as ATBF1. There is at least another SUMOylation site in the F4 fragment of ATBF1, but we were unable to identify this site using the same approaches used for the other sites (Fig. 4), because the F4 fragment contained 29 lysine residues, none of which was located in the consensus SUMOylation motif.

SUMOylation of ATBF1 is dependent on its nuclear localization, because interruption of its nuclear localization prevented its SUMOylation in cells but had no effect on its SUMOylation in a cell-free system (Fig. 5). Considering that ATBF1 associates with PML NBs and that PML NBs play an important role in protein SUMOylation, it is possible that PML NBs also mediate the SUMOylation of ATBF1, but the mechanism of ATBF1 SUMOylation is currently unknown. The PIAS3 SUMOylation E3 ligase was previously shown to interact with ATBF1 [38], but PIAS3 interrupted rather than enhanced ATBF1 SUMOylation (Fig. 6), indicating that PIAS3 is not the SUMO1 E3 ligase that mediates ATBF1 SUMOylation. The SUMO E3 ligase for ATBF1 SUMOylation, if it exists, is currently unknown.

ATBF1 plays a role in multiple biological processes such as gene transcription, development and tumorigenesis. It is certainly possible that SUMOylation of ATBF1 is important for its functions in these processes, as suggested by the fact that ATBF1 SUMOylation depended on its nuclear localization and that ATBF1 is often mislocalized into the cytoplasm in cancer cells, although the functional consequence of ATBF1 SUMOylation in different processes is unknown at this time. SUMOylation affects diverse aspects of a target protein, including its cellular localization, its interactions with other proteins, and its protein stability. At present, it is technically challenging to evaluate the effects of ATBF1 SUMOylation on its functions, because there is neither an efficient experimental system for functional studies of ATBF1 nor a SUMOylation-deficient mutant of ATBF1 for such studies.

In summary, we have characterized the nuclear localization and SUMOylation of ATBF1 in epithelial cells using biochemical approaches and cellular microscopy. In addition to confirming the nuclear localization of ATBF1 in epithelial cells, we identified the NLS for ATBF1, and found that ATBF1 concentrated to form nuclear body-like dots, some of which overlapped or closely associated with PML NBs. Possibly relating to the commonly recognized SUMOylation function of PML NBs, ATBF1 sequestered SUMO1 into the ATBF1 dots, altering the distribution of SUMO1 in the nucleus. Interestingly, ATBF1 itself was SUMOylated at more than three lysine residues, and the SUMOylation depended on its nuclear localization. Finally, the PIAS3 SUMO1 E3 ligase, which directly interacts with ATBF1, diminished rather than enhanced ATBF1 SUMOylation and prevented the co-localization of ATBF1 with SUMO1 in the nucleus. Taken together, our findings suggest that SUMOylation in the nucleus is important for ATBF1 functions, and that ATBF1 could cooperate with PML NBs to regulate protein SUMOylation in different biological processes.
